# Robotic-assisted bronchoscopy—advancing lung cancer management

**DOI:** 10.3389/fsurg.2025.1566902

**Published:** 2025-06-03

**Authors:** Aliss T. C. Chang, Joyce W. Y. Chan, Ivan C. H. Siu, Wei Liu, Rainbow W. H. Lau, Calvin S. H. Ng

**Affiliations:** Division of Cardiothoracic Surgery, Department of Surgery, Prince of Wales Hospital, The Chinese University of Hong Kong, Hong Kong SAR, China

**Keywords:** lung cancer, robotic-assisted biopsy, electromagnetic navigation bronchoscopy, multidisciplinary approach, robotic bronchoscopy, hybrid operating room, transbronchial ablation

## Abstract

The incidental discovery of early-stage, multifocal lung cancer is transforming the medical landscape. Diagnosing and treating such lesions are often troublesome due to their small size, subsolid consistency, and multifocal nature. This has led to the development of electromagnetic navigation bronchoscopy, which enhanced the ease of navigation and improved localization accuracy during diagnostic procedures. Moreover, it opens the door for intricate transbronchial therapeutic procedures thanks to the superior navigational precision. To further automate navigation and increase maneuverability, robotic-assisted bronchoscopy was developed in recent years, where the robotic arms allow a high level of control and stability of the bronchoscope. Recent evidence has shown that the maneuverability, steadiness, and localization accuracy offered by robotic-assisted bronchoscopy systems with the navigation system allow operators to navigate narrower airways and perform complex interventions with great precision. This review illustrates the development, advantages, and applications of various robotic bronchoscopy systems with the latest evidence. We explore the promising future of robotic-assisted bronchoscopy, where such procedures are anticipated to play an essential role in the multidisciplinary management pathway.

## Introduction

Lung cancer ranks as one of the most prevalent cancers in the world and is one of the leading causes of cancer-related mortality worldwide (1). In recent decades, the incidental detection of small, subsolid, multifocal lung nodules has increased significantly, primarily due to the widespread use of low-dose computed tomography (LDCT). This trend is particularly notable following the introduction of evidence-based screening programs for high-risk populations, supported by large randomized control trials such as the NELSON, NLST, and TALENT trials (2–4). These small lung nodules often exhibit pre-malignant or early malignant characteristics and, therefore, should not be overlooked (5, 6) as the prognosis of lung cancer is closely linked to the stage at which it is diagnosed (7). However, diagnosing and treating them can be challenging because of their size and subsolid nature (8–10).

To address these challenges, several technologies have been developed to enhance diagnostic accuracy and treatment effectiveness of lung cancer, especially in early and multifocal lung cancer. These include conventional flexible bronchoscopy and radial probe endobronchial ultrasound (rEBUS). A notable advancement is the use of electromagnetic navigation bronchoscopy (ENB) in diagnostic procedures, along with transbronchial thermal ablation therapy, have transformed the management of multifocal lung malignancies (11). Although ENB excels in localization accuracy through the integration of reality imaging and electromagnetic (EM) guidance, its handling stability and maneuverability require further optimization. Following the trend of robotic-assisted thoracic surgeries, robotic-assisted bronchoscopy (RAB) has emerged to augment navigation accuracy and bronchoscopic maneuverability. RAB shows excellent potential for diagnostic and therapeutic procedures for lung nodules, including transbronchial biopsy and microwave ablation. Moreover, its ability to facilitate comprehensive one-stop treatment for multifocal lung cancer in a hybrid operating room (HOR) is an exciting prospect for future advancements in this field. The prospect of RAB leading to shorter procedural time and improved overall outcomes underscores its importance as a tool for future advancements in lung cancer management. As technology advances, RAB is poised to play a crucial role in revolutionizing lung cancer treatment, potentially transforming the standard of care in lung cancer.

This review will provide an overview of the development of RAB, highlighting its applications, benefits, and discuss future innovations of RAB in lung cancer management with insights from relevant literature.

## The lead up to development of robotic-assisted bronchoscopy (RAB)

Since the 1960s, when the first flexible fibreoptic bronchoscope was pioneered, flexible optical bronchoscopy has been a pivotal tool for managing lung cancer, from diagnosing different pathologies to various therapeutic interventions. Technologies such as videoscope capability, angulated endoscope, and radial probe endobronchial ultrasound (rEBUS) were gradually introduced to improve the ease of operation, localization accuracy, and diagnostic yield (12). With advancements in bronchoscopic technology, transbronchial techniques may offer a lower risk of complications than conventional transthoracic techniques, such as those in percutaneous biopsy and percutaneous radiofrequency ablation (13–15). The low complication rate is primarily due to avoiding direct visceral pleura puncture and, therefore, reduced rate of pleural-based complications, especially pneumothorax (16, 17). Additionally, the transbronchial approach allows accessibility to a broader area of the lungs with fewer anatomical constraints, which can be a limitation of the percutaneous approach (18). However, despite these developments, the diagnostic yield and localization accuracy for lung cancer remain mediocre, especially in small, peripheral lung lesions (i.e., located in the outer one-third of the lungs). A previous meta-analysis showed a pooled diagnostic yield of 70% in guided-bronchoscopy technologies (19). Another systemic review of 35 studies found a diagnostic rate of 88% in central lesions. However, the diagnostic rate was only 78% in peripheral lesions, and in lesions less than 2 cm in size, the diagnostic rate was only 34% (20). A prospective trial published in 2018 comparing conventional bronchoscopy with or without rEBUS in lung nodule biopsy showed a suboptimal diagnostic yield of 49% and 37% in both comparison arms (21). Some investigators reported a low diagnostic yield of 14% in peripheral lesions less than 2 cm in size when using conventional fiberoptic bronchoscopy (8). This marginal diagnostic and localization accuracy can be explained by difficulty maneuvering into smaller and higher generations of airways, the lack of direct visualization, the inability to guide the instrument directly to the lesion, the struggle to maintain stability during instrument exchange due to respiratory motion, and operator navigation error in the distal airways.

Electromagnetic navigation bronchoscopy (ENB) is a dedicated system that improves localization accuracy. It employs EM positioning and specialized software to analyze the preoperative computed tomography (CT) and format the CT images into a 3D roadmap of the tracheobronchial system (22). This 3D roadmap creates a virtual pathway toward the lesion, and the operator can manually guide the bronchoscope along the planned pathway. Similar to other transbronchial approaches, it offers a lower rate of complications due to the avoidance of pleural puncture (23). Moreover, the EM navigation allows real-time tracking of the bronchoscope position during the procedure. The locatable guide and extended working channel enable the operator to guide the instrument directly to the lesion under EM guidance, ultimately enhancing navigational accuracy (24). In the NAVIGATE Study, a multicenter, single-arm study of the SuperDimension ENB system, including 1,157 patients who underwent ENB-guided biopsy, the localization success rate was 94% while the diagnostic yield was 73%, with close to 50% of the lesions being less than 2 cm in size (25). A similar result was replicated in another multicenter study, which included 479 patients with a median lesion size of 2 cm. The overall diagnostic yield was 74.9% (26). Regarding the utilization of ENB in lung lesion localization, previous studies reported a high localization success rate of 90%–100% while having a shorter procedural time and fewer complications compared to the traditional percutaneous approach (27–29).

While ENB transforms the navigational capability in the bronchial tree, the challenges in maneuverability and handling stability during bronchoscopic procedures remain unresolved. This led to the introduction of RAB to further automate bronchoscopy and improve the ease of navigation. In addition to the EM navigation, RAB utilizes robotic articulating arm(s) to manipulate the flexible bronchoscope, and the operator controls the robotic arm(s) remotely using a remote control similar to a gaming control. With the robotic arm(s), the operator can advance the bronchoscope steadily into narrower and smaller distal airways up to the 9th generation (30, 31). Not only does it allow excellent maneuverability to the more peripherally located lesion in the lungs, but it also allows more complex interventions to be performed due to the increased stability during handling and the exact spatial orientation that it provides (31). The combination of EM guidance and robotic arm offers better ergonomics for the operator, expanded accessibility to different regions of the lungs, enhanced stability for interventional procedures, and, ultimately, improved localization accuracy with superior diagnostic yield (32, 33).

## Different RAB platforms

Currently, the US Food and Drug Administration (FDA) approves three robotic bronchoscopy platforms: the Monarch robotic Endoscopy System by Johnson and Johnson, the Ion Endoluminal System by Intuitive Surgical, and the Galaxy System by Noah Medical. These systems differ regarding their bronchoscope design and navigation technology, which we shall elaborate on.

### Monarch endoscopy system

**Figure 1:**
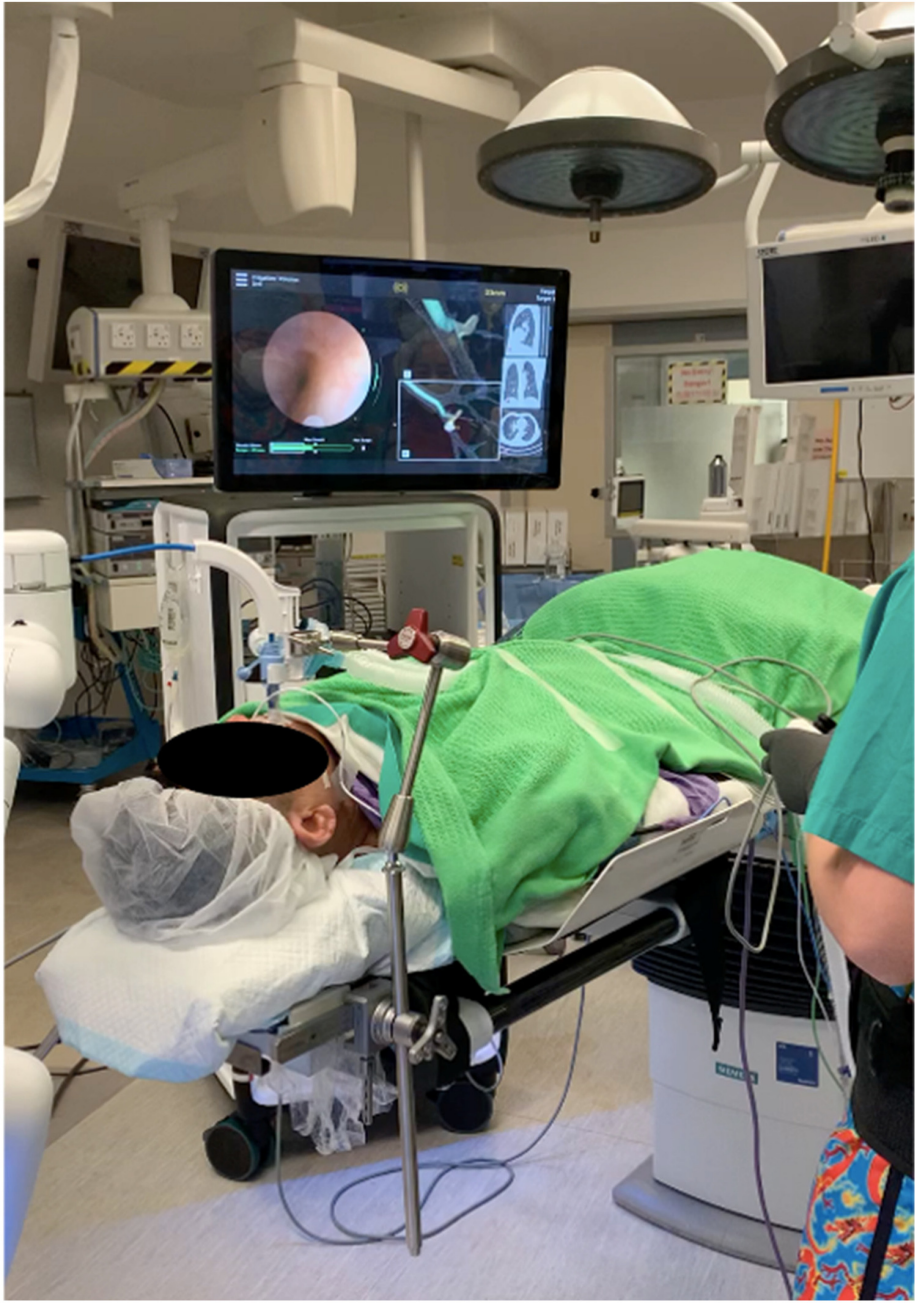
Navigation was performed with the monarch system. The conventional bronchoscopic view was displayed on the left side of the main screen. The 3D reconstructed roadmap with a virtual bronchoscopic pathway was displayed on the right side of the main screen. The operator can use the virtual path to guide the bronchoscope into a narrower airway.

The Monarch Endoscopy System, developed by Auris Health (later acquired by Johnson and Johnson) in California, USA, was the first robotic bronchoscopy platform approved by the US FDA in 2018. It consisted of an inner articulating 4-way 180° steering bronchoscope (4.2 mm outer diameter) and an outer sheath (6 mm diameter). The outer sheath provides structural support during the procedure to enhance stability, while the inner bronchoscope is highly steerable to improve maneuverability (34). This platform uses EM navigation with an external EM field generator and reference sensors on the patient's chest, which can recognize the bronchoscope tip position within the lungs (35). Throughout the navigation process, continuous feedback on the location of the scope tip and the distance from the target is provided. Like ENB, a pre-procedural CT is used to pre-plan a desired pathway toward the target lesion. With the assistance of EM guidance, virtual bronchoscopy, and conventional bronchoscopic vision, the bronchoscope can be guided along the planned pathway using the robotic arm (Figure 1).

### Ion endoluminal system

The Ion Endoluminal System, developed by Intuitive Surgical in California, USA, was approved by the US FDA in 2019. This system has a thin, flexible 180° robotic catheter (3.5 mm outer diameter), with a 1.7 mm vision probe inserted via the working channel during navigation to provide conventional bronchoscopic vision. The entire length of the robotic catheter is equipped with shape-sensing fibers, and this system uses shape-sensing technology to provide real-time shape and location feedback for navigation by sensing the degree of catheter deformation (36, 37). By reconstructing the pre-procedural CT images, a virtual bronchial roadmap is generated. The shape-sensing technology will transmit information about the motion of the catheter and correlate the catheter tip position with the virtual roadmap to provide constant information about the catheter tip position, target location, and distance from the target. Once the navigation is confirmed, the vision probe is removed to allow instrument insertion for interventional procedures. Hence, a live bronchoscopic view is precluded during the procedure. The Ion System does not rely on EM guidance, thus nearby metal objects will not interfere with it, and no special room mapping is required.

### Galaxy system

**Figure 2:**
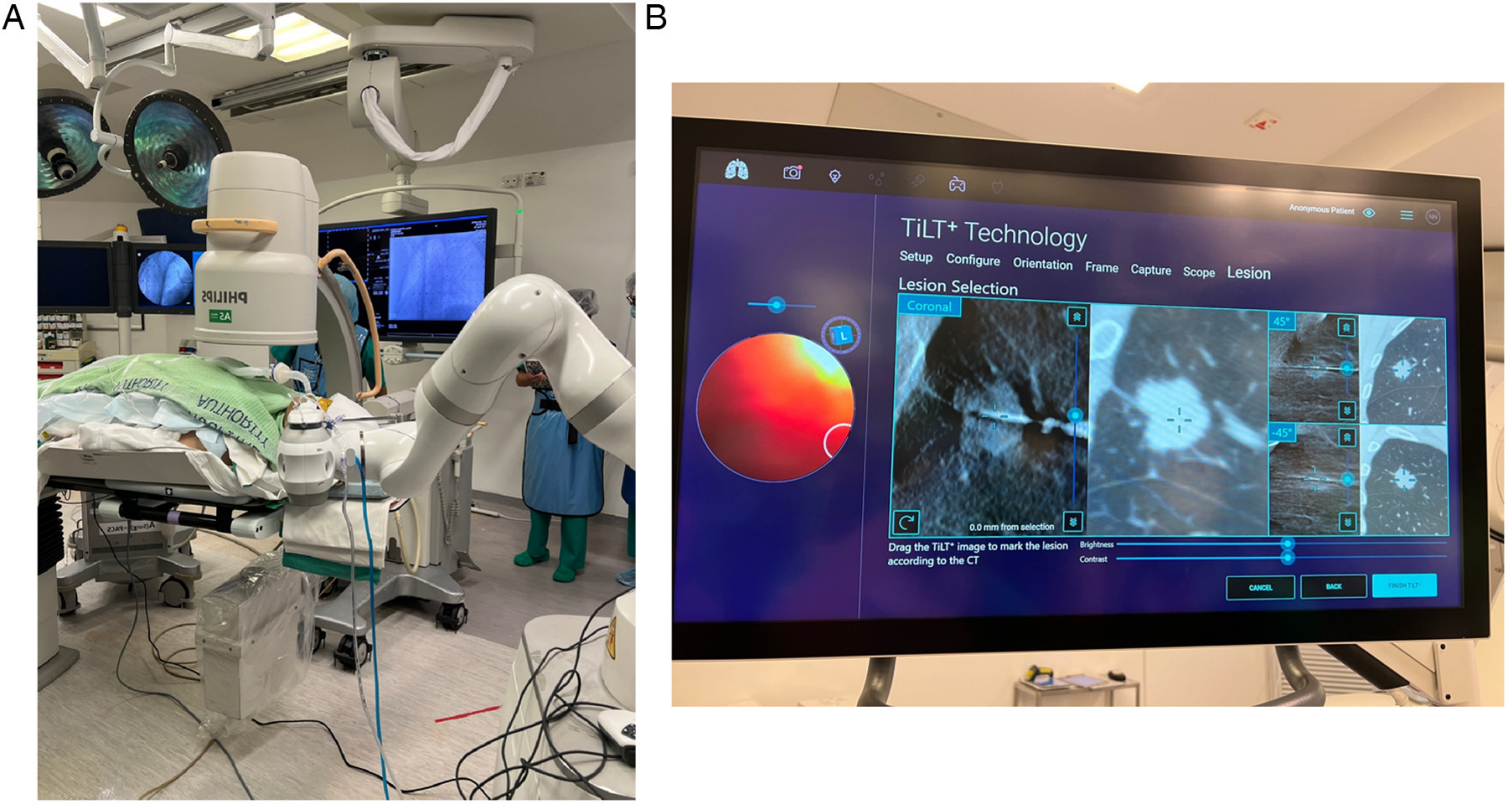
**(A)** The Galaxy System with standard C-arm. **(B)** Tool-in-lesion tomosynthesis (TiLT) technology updates the target location using images from the mobile C-arm and an integrated digital tomosynthesis.

The Galaxy System, developed by Noah Medical in California, USA, was the newest robotic system approved by the US FDA in 2023. It comprises a disposable single-use bronchoscope (4 mm outer diameter) and an EM navigation system. Like other ENB systems, it navigates using a 3D reconstructed roadmap built from a pre-procedural CT. In addition, it has the tool-in-lesion technology (TiLT), which combines EM navigation with integrated digital tomosynthesis and augmented fluoroscopy to allow correction of CT-to-body divergence, which is the discrepancy between pre-procedural static CT images and the dynamic real-time intra-procedural position of lesion resulting from ventilating lungs. Digital tomosynthesis is performed using a fluoroscopy C-arm to capture a series of x-ray images from various angles to reconstruct a 3D image, which is then used to expose the target lesion and provide local registration adjustment based on the updated lesion location (Figures 2A,B).

#### Up-and-coming RAB systems

**Figure 3:**
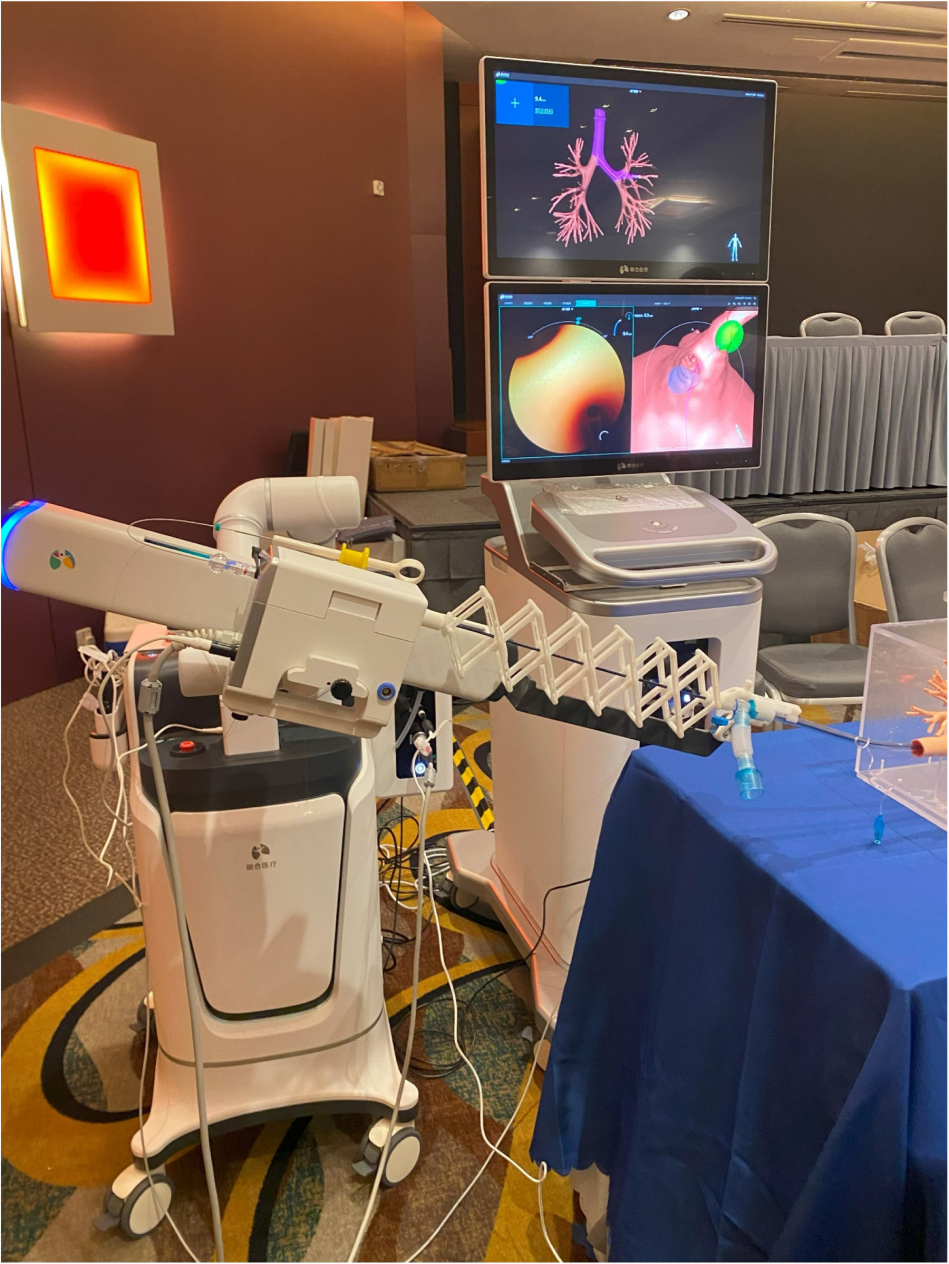
The Unicorn™ RAB System, developed by LungHealth MedTech, China.

Apart from the FDA-approved RAB systems mentioned above, newer systems are emerging and under constant investigation. For example, the Unicorn™ RAB System, developed by LungHealth MedTech in Shanghai, China, is a rising robotic-assisted bronchoscopy system. It utilizes EM navigation with a flexible robotic-articulated bronchoscope held by two articulating robotic arms. A closed-loop actuator makes the precise motion of the bronchoscope possible, allowing the distal end of the robotic tool to rotate 360° and bend at a maximum angle of 200° in all directions. Hence, the Unicorn System permits navigation to peripheral airways up to the 10th generation, benefiting from the highly flexible distal tip (Figure 3). Further clinical study is needed to examine this new system's operability and clinical efficacy.

## Utilization of RAB in lung cancer treatment

One of the original uses of RAB is for diagnostic purposes. Hypothetically, RAB can achieve a higher diagnostic yield when compared with other transbronchial biopsy techniques, thanks to the steady robotic arms offering better maneuverability and stability even in narrower bronchioles. Various studies in recent years have been in support of this. Firstly, a cadaveric study on the diagnostic yield of RAB biopsy was conducted using the Monarch system in 2020, which showed a 97% diagnostic yield in artificial peripheral tumors ranging from 1 to 3 cm in size (38). Likewise, the Precision-1 study compared the localization rate and diagnostic yield of rEBUS, ENB, and the Ion Endoluminal System in artificially implanted small peripheral nodules in cadavers. Results showed a higher rate of successful puncture of nodules in RAB of 80% compared with 45% with ENB and 25% with rEBUS (39). Following the encouraging results from these cadaveric studies, more clinical studies utilizing various robotic systems were conducted, showing positive results in diagnostic yield and feasibility. The PRECIsE study was a prospective multicenter study published in 2021 that included 67 nodules biopsied using the Ion Endoluminal System, with a median maximum diameter of less than 2 cm, and achieved a biopsy completion rate of 97% without pleural-based complications (40). Subsequently, the first prospective multicenter pilot and feasibility (BENEFIT) study using the Monarch System was conducted that included 54 patients, with a lesion localization success rate of 96.2%, and pneumothorax occurring in 3.7% of cases (2 cases) (33). Eventually, a larger multicenter prospective (TARGET) trial of the Monarch System was presented in 2024, consisting of 679 study subjects, with a median lesion size of 1.85 cm and diagnostic yield of 63.8% (using strict methodology), 76.6% (using intermediate methodology), and 87% (using liberal methodology). In the study the sensitivity of malignancy was above 81%, and a low rate of adverse events was reported at 3.8% (41). All the above findings demonstrate a satisfactory localization rate and diagnostic yield while verifying an excellent safety profile with RAB biopsy. Crudely comparing the published data on conventional bronchoscopic, ENB, and RAB biopsy, the RAB safety profile and diagnostic yield are superior (42). Nonetheless, it is essential to note that there is no clinical study to date that directly compares the different biopsy approaches, and there is no randomized controlled trial comparing different robotic platforms or comparing RAB to the traditional percutaneous transthoracic approach. Well-designed trials would be essential to better compare these different approaches (43).

A recent meta-analysis of RAB in diagnosing peripheral lung lesions was published in 2024 by Dr Zhang et al. Ten studies with a total of 724 lesions were included in this meta-analysis. The pooled diagnostic yield was 80.4%. The pooled diagnostic yield in lesions smaller than 2 cm was 78%, grossly higher than those reported in conventional bronchoscopic or ENB biopsy (44). Despite a satisfactory pooled diagnostic yield reported in this analysis, the heterogeneity in diagnostic yield among clinical studies was observed, ranging from 70% to 90%. This may be partly explained by the presence of CT-to-body divergence, which, to overcome this issue, various imaging adjuncts have been applied for tool-in-lesion confirmation (45, 46).

**Figure 4:**
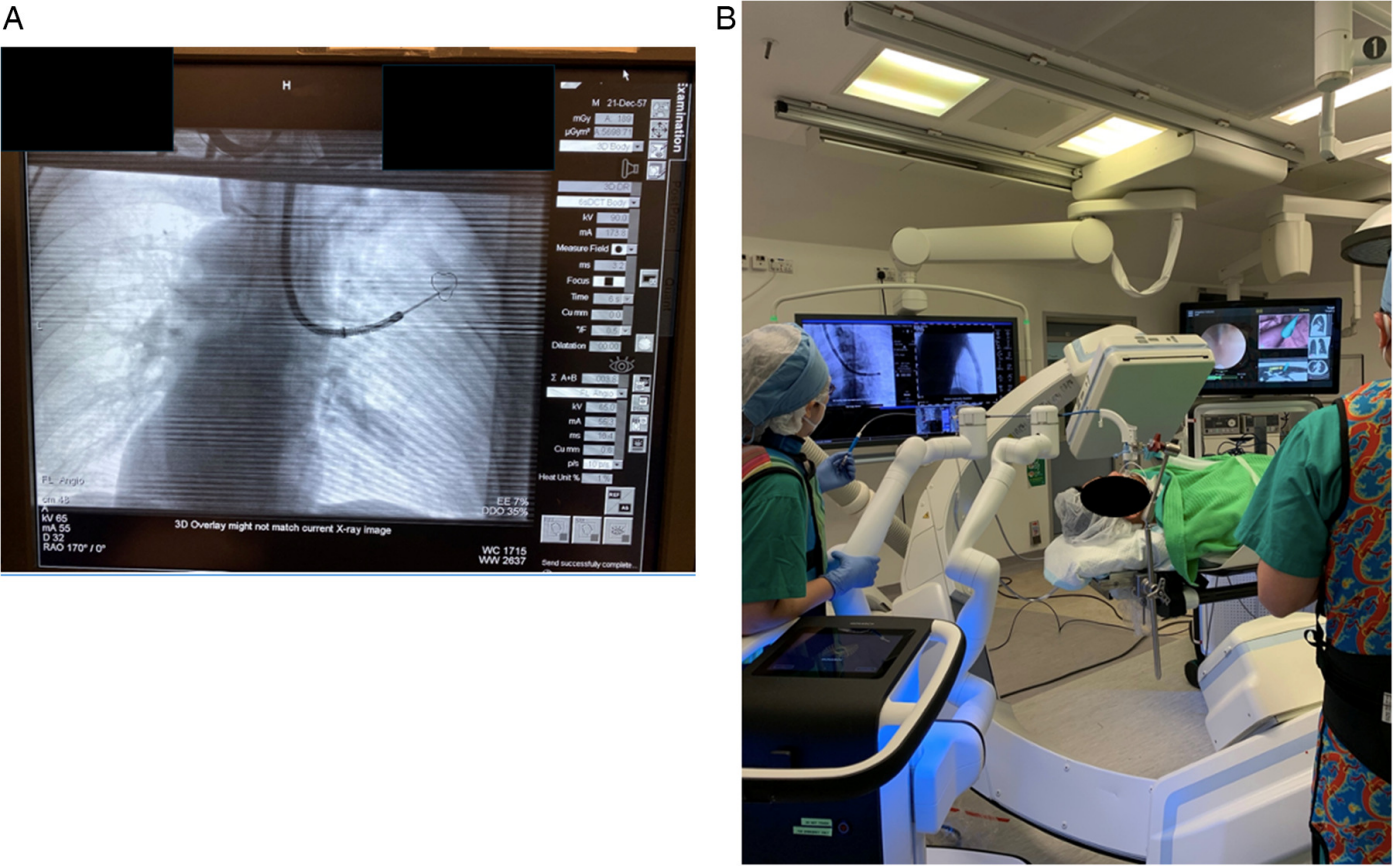
**(A)** Combining multimodal imaging in the HOR increases navigation accuracy and confirms the tool-in-lesion position. Real-time fluoroscopy was used to confirm the tool position during the RAB navigation. **(B)** Subsequently, the patient was kept in the same position, and CBCT spin was performed to confirm the tool-in-lesion position.

Due to the discrepancy between pre-procedural CT done in a static lung with breath-holding and intra-procedural CT done in ventilating and dynamic lungs, there is an expected discrepancy in the actual location of the target lesion, and this is termed “CT-to-body divergence” (47). Various adjuncts were introduced to ensure tool-in-lesion position during RAB procedures under the influence of CT-to-body divergence, including the combined use of rEBUS, real-time fluoroscopy, tomosynthesis, mobile CT, cone-beam CT (CBCT), and multimodal imaging in the HOR (Figures 4A,B). Kalchiem-Dekel et al. reported the concomitant use of rEBUS and fluoroscopy with the Ion Endoluminal System resulted in an 81.7% diagnostic yield and a 98.7% navigational success rate in 159 lesions (48). Pritchett and his colleagues also presented their retrospective cohort using the Ion Endoluminal System concomitantly with rEBUS and CBCT in 2021. Among the 230 lesions, with a median lesion size of 1.5 cm, the overall diagnostic yield was 92.2%. Specifically, the diagnostic yield was 89% in lesions less than 1 cm in size. This cohort validated the outstanding diagnostic yield in smaller lesions using RAB with an ancillary imaging technique (49). Overall, one can conclude from these studies that RABs were conducted using a wide range of ancillary image techniques which seems to be an efficient and replicable method to confirm tool-in-lesion in real-time and can significantly boost the diagnostic yield (50).

Unfortunately, medical resources are sometimes limited, and multimodal imaging might not be available in every scenario. Hence, there is a demand to develop a robotic system with integrated technology to overcome CT-to-body divergence. As of now, the Galaxy System is the only robotic system with integrated tool-in-lesion technology, as described previously, to mitigate CT-to-body divergence. Saghaie et al. reported the first human trial (FRONTIER study) using the Galaxy System in 2024 that included 19 nodules for biopsy with an average lesion size of 2 cm. The localization success rate using TiLT was 100%, and the diagnostic yield was 94.7% (intermediate methodology) and 89.5% (strict methodology) (51). Currently, there is an ongoing clinical trial with an estimated enrollment of 25 patients, evaluating the localization (tool-in-lesion) and diagnostic accuracy of the Galaxy System. This trial is expected to be completed by 2025 (Clinical trial: NCT06056128). The Ion Endoluminal System also has the technology to combat CT-to-body divergence by incorporating the Cios Spin mobile CT. The Cios Spin mobile CT is used to capture CT images during the procedure, which are fedback to the Ion System. 3D imaging reconstruction is created using these intra-procedural CT images, allowing real-time updates on the target location and exact tool-in-lesion confirmation. The preliminary result of an ongoing prospective multicenter (CONFIRM) study on performing biopsy using the Ion Endoluminal System with Cios Spin mobile CT was presented in 2024 by Husta et al. The preliminary analysis included 155 patients with a median nodule size of 14 mm. Tool-in-lesion was achieved in 99.4%, and the strict diagnostic yield was 89%. No pneumothorax was observed among these 155 patients (52). The complete study result is yet to be published (Clinical trial: NCT05562895).

The operator's experience may be a significant factor in achieving reasonable diagnostic and navigational accuracy in bronchoscopic procedures. Interestingly, RAB might have the advantage of a gentle learning curve over conventional bronchoscopy due to the better ergonomics and exact spatial orientation, which enable the operator to master navigation more readily. A multicenter prospective trial was initiated in China to evaluate the learning curve on performing RAB biopsy using the Ion Endoluminal System. The preliminary results were reported in 2022, showing a stable performance was achieved after 18 cases of biopsy, in which the total procedural time and total fluoroscopy time were significantly shortened. Among the 30 nodules biopsied, the diagnostic yield was 90% (53). Another single-center analysis was recently published in 2025, with nine proceduralists performing 551 RAB biopsies. Eventually, six of nine proceduralists achieved proficiency in performing RAB biopsies, and the competency threshold was crossed after 25 biopsies were performed. After the initial learning phase of around 20 biopsies, the operators were observed to start targeting the more challenging nodules with smaller sizes and the lack of bronchus signs (54). These results showed that RAB is a highly achievable procedure in which inexperienced users can get trained with a relatively smooth learning curve.

As the landscape of lung malignancy has shifted after the growing availability of LDCT, where more multifocal and early diseases have been discovered, minimal invasive sublobar lung resection and local ablative treatment have become one of the staples in the management pathway of early-stage and multifocal lung cancer, aiming at preserving lung parenchyma while achieving a reasonable disease control (55). Local ablative therapy in the form of stereotactic body radiation therapy (SBRT) and percutaneous lung ablation has been used in the last decade as an alternative treatment for early-stage lung cancer and oligometastases with decent outcomes (56, 57). However, they also bring several complications, for instance, radiation-related complications such as radiation pneumonitis and pulmonary fibrosis from SBRT (58) and pleural-based complications such as bronchopleural fistula and pneumothorax from percutaneous ablation (59). As bronchoscopic technology evolves, clinicians have now advocated the use of transbronchial ablative therapy for its excellent safety profile and comparable local disease control (60), mainly when performed using navigation bronchoscopic techniques such as CBCT-guided ENB (61–64). To further improve the treatment efficacy of transbronchial lung ablation, RAB is expected to be a reliable platform due to the unparalleled maneuverability and stability that the robotic arm provides. The first animal study of device safety incorporating RAB and microwave ablation by the Neuwave™ Flex Microwave Ablation System was published in 2023. No peri-procedural or post-procedural adverse event was observed in 17 swine models that underwent RAB microwave ablation with CBCT as ancillary imaging (65). With such promising safety results from animal studies, RAB lung ablation is now undergoing ongoing investigation for its clinical capability and safety in human subjects.

**Figure 5:**
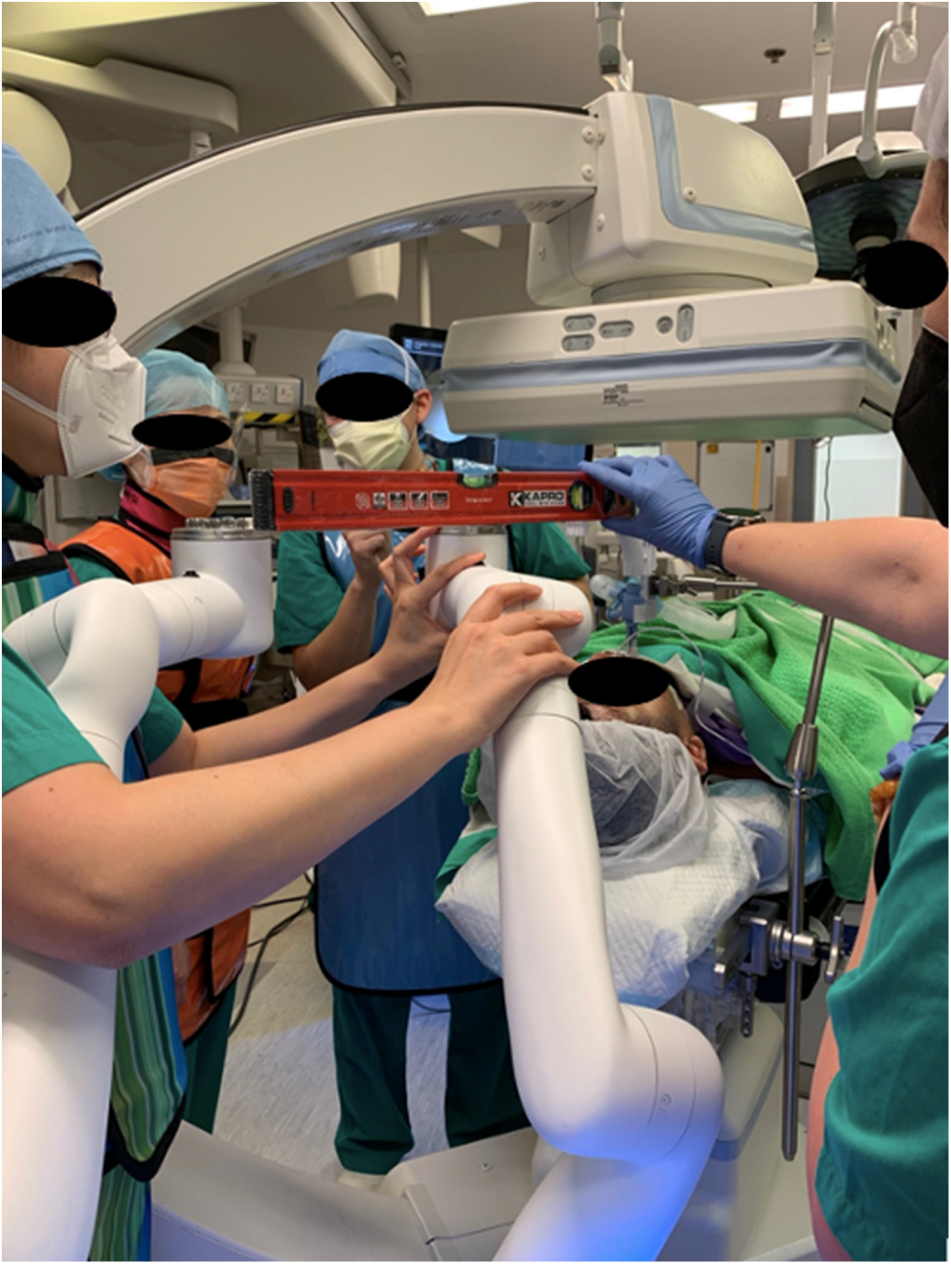
Setting up the monarch system to incorporate the Artis Zeego cone-beam CT (CBCT) as the adjunct imaging in the hybrid operating room (HOR). Careful alignment of the robotic arms is essentially for the accommodation of the CBCT.

The author's institute performed the world's first CBCT-guided RAB microwave ablation for lung metastases in 2022 and has been using RAB in the HOR for diagnostic and therapeutic purposes for years (66). One of the most significant advantages of performing RAB in the HOR is the ability to carry out multiple procedures and offer a streamlined management package for the patient (Figure 5). Thus, it can shorten the total procedural time, avoid repeated general anesthesia (GA) sessions, and treat multiple lesions in one go (67, 68). Assorted combinations of procedures can be performed using RAB. For example, perform RAB biopsy and same session microwave ablation to provide both diagnosis and treatment, or use RAB to dye mark a small lung nodule and perform same session video-assisted thoracoscopic surgical (VATS) resection. Such a technique was reported in 2022 by using the Monarch Robotic System in the HOR. Successful navigation to the target ground-glass opacity was performed, and transbronchial triple dye marking with an additional metallic fiducial marker placement for the resection margin was carried out. Immediately after the localization, a VATS segmentectomy was performed in the same session (69). This technique limits the idle time between lesion localization and the surgery, hence reducing the risk of dye diffusion. Moreover, this workflow also minimizes patient discomfort as this was all performed under one GA session (70, 71).

## Upcoming innovations

RAB is an excellent tool that helps clinicians enhance clinical outcomes and may improve patient's quality of life. Although data on its use have shown favorable results in lung cancer management, more future innovations are needed to refine its utilization and improve diagnostic and therapeutic efficacy.

In previous literature, the diagnostic yield of transbronchial biopsy falls to as low as 30% when the target lesion is parallel or adjacent to the peripheral bronchus (72). Despite the benefits of RAB, this peculiar lesion location remains a challenge for operators in achieving a tool-in-lesion position for the best yield. A steerable biopsy tool was created to enhance the diagnostic yield of RAB biopsy in such cases. A feasibility study in cadavers using steerable biopsy needles was reported in 2023 (Bullseye Study). For the study, a unidirectional steerable biopsy needle was deployed into artificial tumors in the cadaveric model using a conventional bronchoscopy under image guidance (CBCT and fluoroscopy), and fiducial markers were placed within the lesions. The distal tip of the steerable needle permits articulation up to 70° and allows 360° rotation. Among the 15 artificially placed targets, 93.3% of successful marker placement was achieved, and 60% of the markers were placed in the central zone. This study suggests that steerable needles can potentially enhance diagnostic yield by improving the tool-in-lesion rate (73). The higher degree of motion provided by the articulated distal tip offers increased agility and controllability for needle placement, and it allows finer adjustment of the needle tip, which can be guided to different areas of the target lesion to obtain a more representative specimen.

To further optimize operational stability and minimize procedural-related risks, autonomous navigation with artificial intelligence (AI) co-pilot was investigated in recent years. AI co-pilot bronchoscope robot was designed for safer steering within the airways and to enhance navigation accuracy. An AI-human shared control algorithm was developed based on reinforcement learning from experts. This algorithm can predict the operator's steering action, combined with the bronchoscopic images being input into the algorithm, and the co-pilot bronchoscopic robot will automatically keep the tip of the bronchoscope in the center of the airway and reduce operational error. Studies published in 2024 showcasing this novel technology in simulated airway models and live porcine lungs. By detecting image errors using AI, the AI co-pilot was shown to reduce operational errors in all operators with different levels of expertise. Moreover, it allows novice operators to perform bronchoscopic navigation safely and competently. By centering the bronchoscope automatically, the risk of incidental airway injury during the procedure was also diminished (74, 75).

**Figure 6:**
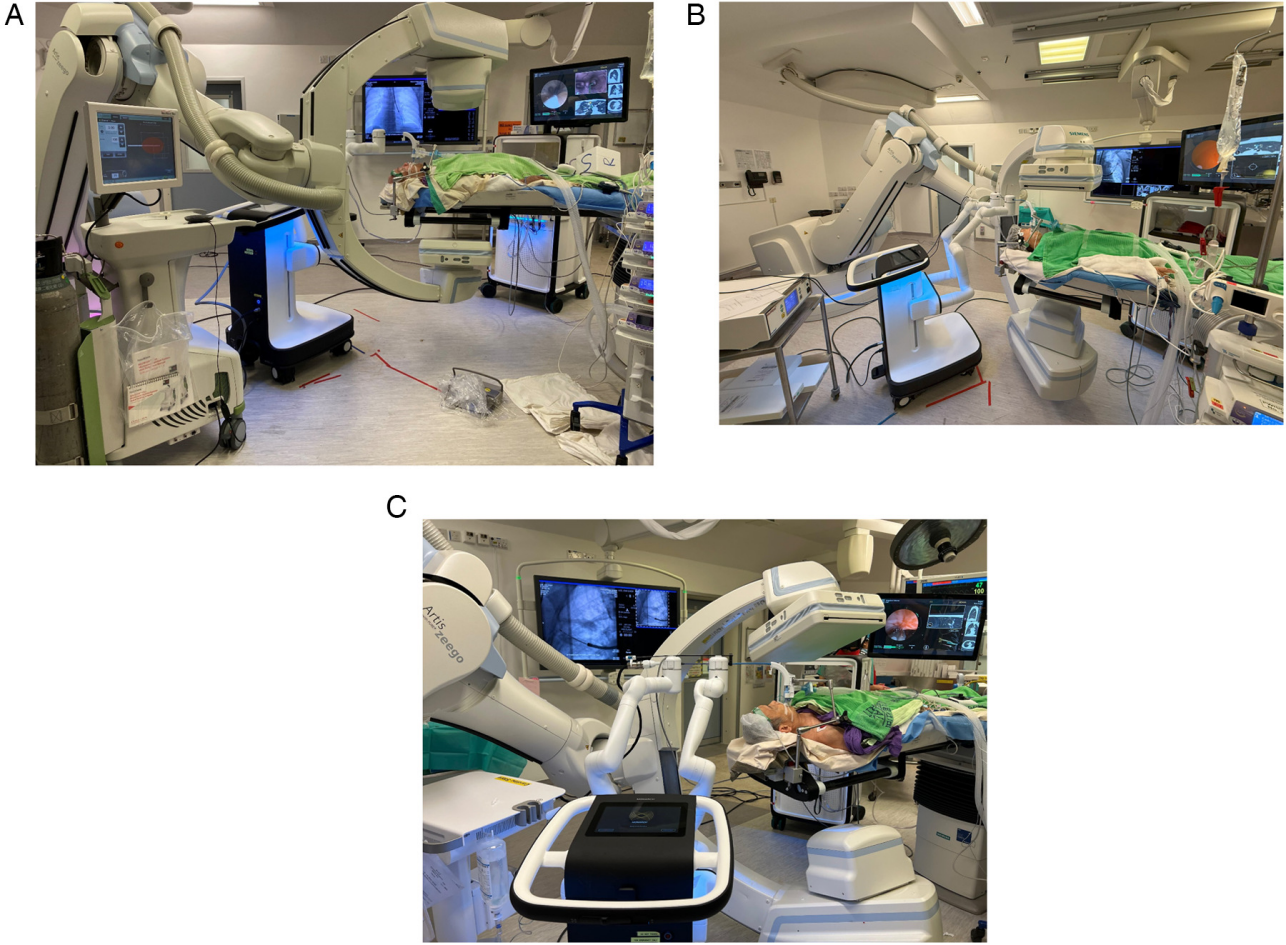
Robotic-assisted bronchoscopy delivering microwave ablation using **(A)** Johnson and Johnson Neuwave™ Flex catheter; **(B)** Medwaves AveCure® catheter and system; and **(C)** Medtronic Emprint™ catheter and system.

Regarding therapeutic interventions, various ablative therapies are continuously investigated for their uses with RAB. Performing ablative therapy using the RAB systems has the advantage of increased navigational accuracy to guide the ablation catheter to peripheral lesions, which would otherwise be difficult to navigate to. Also, RAB can provide a stable platform during the energy delivery from an ablative catheter source (Figures 6A–C). From earlier clinical studies, microwave ablation is a well-established energy modality to be used with navigation bronchoscopy; other types of transbronchial ablative therapy are emerging, such as cryoablation (76), radiofrequency ablation with microperfusion (77), laser, vapor steam ablation (78), and pulsed electric field (PEF) [Clinical trial: NCT05890872, (79)]. The different types of energy modality each have their unique mechanism of producing energy and, therefore, will produce ablation zones with distinctive characteristics. From a clinical perspective, performing therapeutic ablation with these energies with RAB is possible, as many of them are compatible with navigation bronchoscopy. However, the compatibility, feasibility, and safety of performing various ablative therapy using RAB systems remain to be determined, and further evidence from more extensive prospective trials is needed. Transbronchial photodynamic therapy (PDT) is also an up-and-coming therapeutic option for malignant lung or endobronchial lesions. It is done by delivering a cancer-specific photosensitizer to the target lesion and using a light source with a specific wavelength to stimulate free-radical generation within the cancer cells, thereby destroying them. Recently, investigators have reported the utilization of transbronchial PDT in peripheral lung cancers in a phase 0 trial, which showed no significant acute complication. Still, the treatment effect was suboptimal due to the low light dose (80). Hence, more clinical studies on PDT, especially when combined with RAB, are expected. Another alternative therapeutic procedure that RAB can potentially deliver is the intratumoral injection of chemotherapy and immunotherapy agents. RAB's exact spatial orientation and increased stability allow explicit needle placement for agent injection. The precise injection can deliver a therapeutic agent directly to the tumor microenvironment to augment the therapeutic effect while reducing the risk of systemic toxicity (81). Intratumoral placement of radiation seeds and radioenhancers are inventive treatment options that can be delivered via the transbronchial route. Radiation seeds, such as the Alpha DaRT™s, can emit alpha radiation locally to the tumor cells and act as local radiation therapy to the tumor. Because alpha radiation has a short transmission range in soft tissue, local radiation therapy is expected not to affect other distal organs and, thus, produce fewer systemic side effects compared to SBRT. A feasibility and safety study conducted using the Alpha DaRT™s technology was published in 2024 with swine models. One-hundred and fifty-eight Alpha DaRT™s were successfully delivered using a bronchoscopic approach into lung parenchyma. No change in general condition was observed in the swine after the implantation, and hematological evaluation showed no treatment-related abnormality. No significant migration of Alpha DaRT™ was reported (82). This study demonstrated the feasibility of bronchoscopic delivery of radiation seed. For clinical application in humans, an active clinical trial is underway to evaluate the use of Alpha DaRT™s in recurrent lung cancer (Clinical trial: NCT05632913). On the other hand, a radioenhancer can also be injected intratumorally by bronchoscopic route to up-regulate radiation sensitivity of the tumor cells. Hence, it can trigger significant cell death in the injected tumor after radiation exposure and ensuing an adaptive immune response within the tumor cells. The CONVERGE study is a recruiting phase 2 randomized clinical trial that studies the treatment outcomes of using radioenhancer in combination with concurrent chemoradiation followed by Durvalumab in locally advanced or unresectable stage 3 lung cancer. Results are expected to be reported in 2028 (Clinical trial: NCT06667908).

Like other new technologies, cost is a significant drawback of RAB and other robotic technologies due to the expensive robotic system and its single-use consumables. The high costs associated with RAB are compounded when integrating expensive imaging techniques, such as CBCT, which are often employed to enhance navigation accuracy but can significantly increase procedural expenses. Currently, there is a notable lack of research evaluating the cost-effectiveness of RAB. As RAB technology advances and becomes more widespread, assessing its economic impact will be essential, particularly in low-income socioeconomic settings, where healthcare resources are more limited. With the rising detection of incidental pulmonary nodules through widespread LDCT imaging, this democratization of advanced diagnostic tools holds promise for improving early detection and management of lung lesions, but also emphasizes the need for future studies to evaluate the balance between clinical benefit and economic sustainability.

## Conclusion

Robotic-assisted bronchoscopy brings exceptional maneuverability and stability to diagnostic and therapeutic lung cancer procedures. This cutting-edge technology proved its ability to enhance diagnostic yield and open up an increasing number of therapeutic options while maintaining a low complication rate. With RAB, one-stop management combining diagnostic, staging, and therapeutic procedures within a single operative session becomes even more feasible, shortening overall procedural time and minimizing patient discomfort. Soon, we can foresee the expansion of the use of RAB globally, paving the way for novel lung cancer therapeutic strategies, such as thermal ablation therapy, PEF treatment, and precision intratumoral treatments.
